# Toxin of Tuberculosis Bacillus

**Published:** 1898-09

**Authors:** 


Toxin of Tuberculosis Bacillus. Sciola (*Rev. Gen. l'Antisep.*) claims to have isolated a special toxic substance from the bacillus of tuberculosis by the action of ether upon glycerin extract. This substance injected in doses of 0.5 c.c. in the veins of a rabbit produces violent convulsions, and was fatal in doses of 2 c.c. In guinea-pigs convulsions of a milder degree were produced. The lethal action of this material is destroyed by a temperature of 100° C.; but a portion of the material which produces the convulsions remains unaffected.—*Western Druggist.*

Diiodide Acetylene. This new compound occurs in colorless needle-like crystals, having a very disagreeable odor; it is volatile, and is soluble in all the ordinary solvents. On exposure to light it assumes a reddish color, and it is lethal to all forms of micro-organisms, and thus it prevents putrefaction and fermentation.

